# Factors Associated with Early Childhood Caries in Polish Three-Year-Old Children

**DOI:** 10.3290/j.ohpd.a45088

**Published:** 2020-09-04

**Authors:** Dorota Olczak-Kowalczyk, Dariusz Gozdowski, Urszula Kaczmarek

**Affiliations:** a Professor, PhD, Department of Paediatric Dentistry, Medical University of Warsaw, Poland. Concept, wrote the manuscript, contributed substantially to discussion.; b Associate Professor, PhD, Department of Experimental Statistics and Bioinformatics, Warsaw University of Life Science, Warsaw, Poland. Performed statistical evaluation.; c Professor, PhD, Department of Conservative Dentistry and Pedodontics, Wroclaw Medical University, Wroclaw, Poland. Concept, wrote the manuscript, contributed substantially to discussion, proofread the manuscript.

**Keywords:** early childhood caries, dmft, oral hygiene

## Abstract

**Purpose:**

To assess the prevalence and associated factors with early childhood caries (ECC) in a Polish population.

**Materials and Methods:**

A cross-sectional study was carried out involving 656 three-year-old preschool children of both sexes. Data were collected through oral examination of the children and a questionnaire self-reported by their parents. The questionnaire contained information on sociodemographic aspects, feeding and oral hygiene practices, dental care utilisation and dental health knowledge. Associations between ECC and caries-related factors were analysed with use of bivariate and multivariate logistic regression and Mann–Whitney U test.

**Results:**

ECC was diagnosed in 64.0% children from the rural area and 46.6% from the urban one, more often in boys (57.7%) compared to girls (49.5%) and S-ECC in 37.1%, 24.2%, 31.5% and 27.5%, respectively. The associations between caries experience and living in a rural area, male sex, education level and oral health-related knowledge of a parent, tooth brushing frequency, nocturnal bottle-feeding and feeding with sweet beverages at the age over 12 months, consumption of sweetened within the first 2 years of age and drinking of sweet beverages once a week at bivariate level were found. In the final model of the logistic multivariate regression analysis, seven variables were associated with ECC experience. They revealed the probability in decreasing order: living in a rural area (odds ratios (OR) = 1.90); feeding the child during the first 2 years with sweetened food (OR = 1.77); nocturnal drinking of sweet beverages by the >12-month-old child (OR = 1.73); education level of parent (OR = 1.53); gender – male (OR = 1.48); nocturnal bottle-feeding of the over-12-month child (OR = 1.44); and frequency of tooth brushing (OR = 1.41).

**Conclusion:**

The most prominent risk factors for ECC were living in a rural area, consumption of sweetened foods within the first 2 years of age and nocturnal drinking of sweet beverages by the over 12-month-old child.

Despite the improvement of oral health in the last few decades, dental caries remains the common chronic disease in childhood. Caries affecting one or more primary teeth and manifesting as non-cavitated or cavitated lesions, missing teeth or filled tooth surfaces due to carious process in a child 71 months of age or younger was defined as early childhood caries (ECC) by the American Academy of Pediatric Dentistry (AAPD). Its severe form (S-ECC) is diagnosed depending on the manifestation and age: in a child younger than 3 years of age any sign of smooth-surface caries, for a child from 3 to 5 years one or more cavitated, missing (due caries) or filled smooth surfaces in primary maxillary anterior teeth, or a decayed, missing, or filled teeth (dmft) score of greater or equal to four at age of 3 years, greater or equal to five at age of 4 years, or greater or equal to six at age of 5 years.^1^

The World Health Organization (WHO) recommends the use of population surveys to assess caries level in primary dentition at the index age group of 5 years (practically between fifth and sixth birthday of children, ie, shortly before start the eruption of permanent teeth).^22^ However, it has been proposed recently during the WHO Global Consultation on Early Childhood Caries the inclusion of the 3-year-olds as one of the index age groups recommended for population surveys in the next edition of WHO Oral Health Surveys: basic methods.^34^

Dental caries prevalence differs between populations as well as within the same population due to interaction of the biological determinants with some confounding factors, which are not always the same in all societies (eg, behavioural and socioeconomic factors). Moreover, being a strongly age-related disease, its prevalence and severity increases with age. The rise in caries prevalence in Swedish children between of 2 and 3 years of age by approximately 11%,^5^ and in English children between 3 and 5 years of age by approximately 20% was noticed.^21^ Moreover, in the same period, the dmft score in English children increased by over 2-fold. ECC prevalence among 3-year-olds ranged from 4% (Sweden, 2011) through 8.6% (Japan, 2016); 11.7% (England, 2013); 13.7% (Germany, 2015–16); 16.8% (Italy, 2010–11); 36.5% (Latvia, 2000); 50.5% (Lithuania, 2010); 58.7% (Bosna and Herzegovina, 2014) to 80% (American Indian, 2016).^21,28,30,32^ In Poland, in 2002, 56.6% children by the age of 3 years were caries-affected and dmft was 2.9.^29^ Polish children, compared to Lithuanian ones, revealed 6% higher caries prevalence and approximately 38% higher dmft value (2.9 vs 2.1)^28,29^ and compared to English children, approximately 45% higher prevalence and approximately 7-fold higher dmft value.^21^

The studies showed numerous relationships between occurrence of ECC and sociodemographic factors, parent’s/guardian’s educational level and knowledge about dental health, cleaning of child’s teeth, utilisation of dental service, feeding practice and established dental home care.^4,6,7,13,17,23,32,33^ However, so far there has been no data regarding comprehensive analysis of ECC modifiable risk factors in the Polish population.

The purpose of the study was to identify the extent to which well-established factors are associated with ECC in Polish population.

## Materials and Methods

The survey was carried out as a cross-sectional national study from October to November 2015. The population study group was selected via a three-stage cluster sampling procedure; in three provinces situated in western, central and eastern parts of Poland, some administrative divisions of second (counties) and third level (communes that are classified as urban and rural) were randomly selected. Then, 33 preschools, both public and private, were randomly chosen. Data on the total number of 3-year-old children were derived from the Central Statistical Office (Demographic Yearbook of Poland 2014). The sample size was calculated based on data concerning caries prevalence in this age group, ie, about 50% of caries-affected children. With such assumptions, and a 95% level of confidence and ± 5% error tolerance, 385 subjects represented a minimum sample size. The protocol of the survey required informed consent from the child’s parent. Requests for signed consent for sampled children along with letters informing about the scope of the study and questionnaires were distributed by the preschool teachers to the parents, who completed the questionnaires at home and returned them to the preschool teachers.

The inclusion criteria were as follows: children attending preschools, which were aged 3 years but below 4 years, present at the time of the survey and subjected to oral examination, as well as with the written consent of the parent and a fully answered questionnaire. The exclusion criteria were: no agreement of preschool authority; the child at the age of less than 3 years and over 4 years; non-cooperative or absent child in the day of the examination; no written consent of a parent; and incompletely filled questionnaire.

The dental examination was performed with the use of artificial light, a plane mirror and ball-ended dental probe (WHO CPI probe). Decayed, missing and filled teeth (dmft) and surfaces (dmfs) were assessed according to the recommended criteria of the World Health Organization.^22^ Prevalence of ECC (dmft ≥1) and severe ECC involving greater than or equal to four smooth surfaces in primary teeth (dmfs ≥4) as well as mean values of dmft and dmfs were calculated.

Assessment of the dental condition was carried out by three teams; each consisted of two examiners being paediatric dentists. The examiners were calibrated before the survey. Each examiner was asked to examine the same group of 14 patients and the findings were compared with those of the experienced supervisor.

The questionnaire covered demographic and social background (gender of the child, area of residence, parents’ education and dental health knowledge, child’s use of dental care, child’s oral health-related behaviours, eg, frequency of tooth brushing, cleaning child’s teeth by a parent, the past and present feeding practices of a child). Oral health knowledge of the parent regarding dental caries was measured using eight items. The questions were as follows: (1) children should regularly attend follow-up visits at the dental surgery; (2) frequent snacking produces favourable conditions for caries development; (3) frequent and excessive intake of sugar causes dental caries; (4) caregivers should help children up to the age of 8 years in teeth cleaning; (5) fluorides can provide protection from dental caries; (6) primary teeth do not require taking such care as permanent ones because they will naturally shed (exfoliated); (7) caries in primary teeth creates favourable conditions for caries development in permanent teeth; (8) bacteria that cause dental caries may be transmitted to the child's oral cavity, eg, by the mother. A score of 1 was given for each correct answer to a question, and a score of zero was given for an incorrect answer or for an answer of ‘I don’t know’. The total score ranged from 0 to 8. The score of 7 or more was assumed a high level of dental health knowledge.

The survey was carried out in the frame of the project ‘Monitoring of oral health condition in Polish population in 2013–2015’ supported by the Ministry of Health (No. 11/1/2015/1210/421 of 18 August 2015). The consent of the Bioethics Committee of the Warsaw Medical University was obtained (KB/216/2015).

### Statistical Analysis

The analysed variables were presented in the form of a percentage or mean with standard deviation. In order to assess the impact of different factors on the prevalence of caries in children, a bivariate logistic regression analysis was conducted in which the impact of each individual factor was considered. Based on logistic regression, odds ratios (OR) were defined for a relative risk of caries development including confidence intervals (with a confidence level of 95%). The variables that remained statistically significant at bivariate level were included in a multivariate logistic regression using the forward stepwise procedure to identify the factors, which were significantly associated with dental caries experience.

Besides, the statistical significance of mean values differences between the two groups was defined with the use of Mann–Whitney U test. The level of statistical significance was set at p <0.05. The analyses were conducted using the SPSS 22, the Statistica 10 and the R 3.2 software packages.

## Results

**Fig 1:**
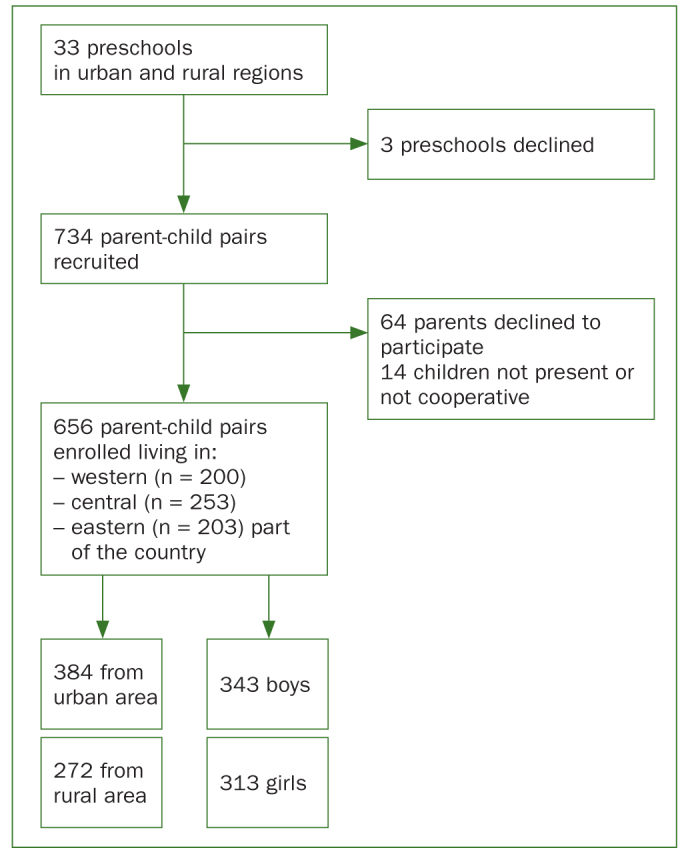
Flow of participants.

The attendance in the survey was voluntary. Approval from the chosen 30 preschool authorities in urban and rural areas was obtained, 15 in every place. 734 children were selected; however, 8.7% (n = 64) of the parents did not response to the request for permission to the study or did not complete the questionnaire, and 1.9% (n = 14) of the sampled children were absent at the time of dental examination or refused to be examined. Therefore, 656 children were finally included in the study (which number covered a minimum representative sample size), out of which 200 were from western, 253 from central, and 203 from eastern parts of the country. They lived in urban (58.5%, n = 384) and rural areas (41.5%, n = 272), and 52.3% (n = 343) of them were males (Fig 1). The majority of parents of the children were reported to have a higher level of education (58.4%, n = 383) and the other secondary (39.5%, n = 259) or primary education (2.1%, n = 14).

The intraexaminer reliability of dental examination was 0.93–1.00 and interexaminer was >0.80 (Cohen’s kappa score).

In the whole sample, ECC occurred in 53.8% of children affecting on average 2.40 ± 3.47 teeth (dmft) and 3.68 ± 7.28 dental surfaces (dmfs). The prevalence of S-ECC (dmfs≥4) was 29.6%.

Bivariate regression analysis regarding sociodemographic variables presented greater likelihood of ECC in children living in a rural area than an urban one (OR = 2.03), in boys than girls (OR = 1.39), having a parent with a lower education level (OR = 1.76) and with lower level of dental health knowledge (OR = 1.41), and attending to dental surgery (OR = 1.36). The relationship of these variables for dmft and dmfs values almost resembled those observed for ECC and although statistical significance was different, the tendency was similar (Table 1).

**Table 1:** Association between caries experience and sociodemographic variables

Variable		ECC	S-ECC	dmft	dmfs
		Prevalence (%) ^a^OR, ^b^95% CI; ^c^p value		Mean ± SD ^d^p value	
Place of residence	Urban (ref)	46.6	24.2	1.97 ± 3.25	2.88 ± 6.25
	Rural	64.0	37.1	2.99 ± 3.68	4.81 ± 8.41
		OR = 2.03, 95% CI = 1.48–2.79; p < 0.001	OR = 1.85, 95% CI = 1.32–2.59; p < 0.001	p < 0.001	p < 0.001
Gender	M (ref)	57.7	31.5	2.64 ± 3.63	4.1 ± 8.04
	F	49.5	27.5	2.13 ± 3.26	3.17 ± 6.31
		OR = 1.39, 95% CI = 1.02–1.89; p = 0.035	OR = 1.21, 95% CI = 0.87–1.70; p = 0.261	p = 0.012	p = 0.013
Parent with higher education level	Yes (ref)	48.0	25.8	2.00 ± 3.04	2.86 ± 5.60
	No	61.9	34.8	2.95 ± 3.92	4.84 ± 9.01
		OR = 1.76, 95% CI = 1.28–2.41; p < 0.001	OR = 1.53, 95% CI = 1.09–2.15; p = 0.014	p <0.001	p <0.001
Parent with high level of dental health knowledge	Yes (ref)	49.7	26.0	2.00 ± 2.99	2.97 ± 5.79
	No	58.3	33.4	2.83 ± 3.88	4.46 ± 8.56
		OR = 1.41, 95% CI = 1.04–1.92; p = 0.028	OR = 1.43, 95% CI = 1.02–2.00; p = 0.038	p = 0.009	p = 0.015
Dental visit	Yes (ref)	57.5	35.2	2.94 ± 4.02	4.92 ± 9.11
	None so far	49.8	23.5%	1.81 ± 2.62	2.34 ± 4.15
		OR = 1.36, 95% CI = 1.00–1.85; p = 0.050	OR = 1.77, 95% CI = 1.26–2.49; p = 0.001	p <0.001	p <0.001

ref – reference category; ^a^OR- odds ratio based on bivariate logistic regression; ^b^95% CI – confidence interval for OR; ^c^p value; ^d^p value; Mann–Whitney U test.

With regard to oral hygiene practice, ECC prevalence was less likely to occur in children brushing teeth twice a day (OR = 1.68) and they had less caries-affected teeth. Teeth cleaning by parents did not affect these parameters (Table 2).

**Table 2:** Association between caries experience and oral hygiene practice

Variable		ECC	S-ECC	dmft	dmfs
		Prevalence (%) ^a^OR, ^b^95% CI; ^c^p value		Mean ± SD ^d^p value	
Tooth brushing twice a day	Yes (ref)	48.4	28.3	2.22 ± 3.40	3.62 ± 7.74
	No	61.2	31.3	2.64 ± 3.55	3.77 ± 6.62
		OR = 1.68, 95% CI = 1.22–2.30; p = 0.001	OR = 1.15, 95% CI = 0.82–1.62; p = 0.407	p = 0.015	p = 0.013
Cleaning child’s teeth by parent	Yes (ref)	56.8	32.3	2.74 ± 3.77	4.56 ± 8.18
	No	52.3	28.2	2.22 ± 3.29	3.24 ± 6.75
		OR = 1.20, 95% CI = 0.87–1.66; p = 0.980	OR = 1.21, 95% CI = 0.85–1.72; p = 0.596	p = 0.185	p = 0.165

ref – reference category; ^a^OR- odds ratio based on bivariate logistic regression; ^b^95% CI – confidence interval for OR; ^c^p value; ^d^p value; Mann–Whitney U test.

Considering past feeding practices, the results showed that ECC was more likely to occur among children at the age of over 12 months fed before or during the sleep with a bottle (OR = 2.23) or sweet beverages (OR = 1.85) as well as during the first 2 years of life fed with sweetened food (OR = 2.38). In addition, a tendency was noticed between ECC occurrence and breastfeeding of children at the age of over 12 months before or during the sleep (OR = 1.40, p = 0.058). The relationship of these factors for dmft and dmfs values almost resembled those observed for ECC and although statistical significance was different, the tendency was similar (Table 3). Children currently consuming sweetened beverages and tea more often than once a week were more likely to have ECC (OR = 1.91 and OR = 1.51, respectively). They also showed tendency to some higher dmft and dmfts values. Although frequent consumption of potato crisps was not a factor influencing ECC occurrence, it could favour more severe caries as dmft and dmfs values were significantly higher in children consuming crisps more frequently compared with those who were not (Table 4).

**Table 3:** Association between caries experience and dietary variables

Variables		ECC	S-ECC	dmft	dmfs
		Prevalence (% ) ^a^OR, ^b^95% CI; ^c^p value		Mean ± SD ^d^p value	
Over 12 month-old child breastfed before or while sleeping	Yes (ref)	59.9	39.0	3.25 ± 4.26	5.54 ± 9.99
	No (neither breast nor bottle fed)	50.0	25.2	1.98 ± 2.96	2.73 ± 4.97
		OR = 1.40, 95% CI = 0.99–1.99; p = 0.058	OR = 1.81, 95% CI = 1.25–2.60; p = 0.001	p = 0.002	p = 0.002
Over 12 month-old child bottle fed before or while sleeping	Yes (ref)	71.4	37.1	3.37 ± 4.10	6.34 ± 12.07
	No (neither breast nor bottle fed)	50.0	25.2	1.98 ± 2.96	2.73 ± 4.97
		OR = 2.23, 95% CI = 1.05–4.73; p = 0.036	OR = 1.44, 95% CI = 0.71–2.91; p = 0.315	p = 0.016	p = 0.018
Over 12 month-old child fed with sweet beverages before or while sleeping	Yes (ref)	64.5	40.2	3.33 ± 4.08	5.37 ± 8.99
	No	47.0	22.8	1.80 ± 2.86	2.61 ± 5.69
		OR = 1.85, 95% CI = 1.33–2.58; p <0.001	OR = 1.87, 95% CI = 1.32–2.64; p <0.001	p <0.001	p <0.001
During the first 2 years of life child fed with sweet food	Yes (ref)	57.1	32.9	2.65 ± 3.64	4.12 ± 7.76
	No	35.9	11.7	1.03 ± 1.82	1.33 ± 2.76
		OR = 2.38; 95% CI = 1.54–3.68; p <0.001	OR = 3.72, 95% CI = 1.99–6.97; p <0.001	p <0.001	p <0.001

ref – reference category; ^a^OR- odds ratio based on bivariate logistic regression; ^b^95% CI – confidence interval for OR; ^c^p value; ^d^p value; Mann–Whitney U test.

**Table 4:** Association between caries experience and current dietary variables

Variables		ECC	S-ECC	dmft	dmfs
		Prevalence (%) ^a^OR, ^b^95% CI; ^c^p value		Mean ± SD ^d^p value	
Sweet beverages once a week	More (ref)	60.0	41.5	3.28 ± 4.33	5.15 ± 9.32
	Less/never	53.1	28.3	2.30 ± 3.35	3.52 ± 7.01
		OR = 1.91, 95% CI = 1.24–2.93; p = 0.003	OR = 2.18, 95% CI = 1.43–3.32; p <0.001	p = 0.134	p = 0.108
Tea sweetened with sugar once a week	More (ref)	57.2	29.8	2.57 ± 3.69	4.07 ± 8.10
	Less/never	47.0	29.0	2.04 ± 2.95	2.90 ± 5.16
		OR = 1.51, 95% CI = 1.09–2.09; p = 0.014	OR = 1.04, 95% CI = 0.73–1.49; p = 0.831	p = 0.066	p = 0.071
Potato crisps once a week	More (ref)	64.2	37.0	3.28 ± 4.19	5.19 ± 9.34
	Less/never	52.3	28.5	2.27 ± 3.34	3.47 ± 6.93
		OR = 1.63, 95% CI = 1.01–2.64; p = 0.260	OR = 1.47, 95% CI = 0.97–2.406; p = 0.118	p = 0.034	p = 0.064

ref – reference category; ^a^OR- odds ratio based on bivariate logistic regression; ^b^95% CI – confidence interval for OR; ^c^p value; ^d^p value; Mann–Whitney U test.

Results from the bivariate logistic regression analysis between sociodemographic, oral hygiene practice and past and present dietary variables showed almost the same likelihood of S-ECC development as for ECC, except for gender, tooth brushing twice a day, bottle-feeding a child at age of over 12 months before or while sleeping, and drinking tea sweetened with sugar (Tables 1–4). In the final model of the logistic multivariate regression analysis, seven variables were associated with ECC experience. They revealed the probability in decreasing order: place of residence – rural (OR = 1.90); feeding the child during the first 2 years of age with sweet food (OR = 1.77); nocturnal drinking of sweet beverages by the over-12-month child (OR = 1.73); education level of parent (OR = 1.53), gender – male (OR = 1.48); nocturnal bottle-feeding of the over-12-month child (OR = 1.44); and frequency of tooth brushing (OR = 1.41). However, S-ECC experience was linked with only five variables at some higher probability. Their arrangement in descending order was as follows: feeding the child during the first 2 years of age with sweet food (OR = 2.96); nocturnal drinking sweet beverages by the over-12-month-old child (OR = 1.95); nocturnal bottle-feeding of the over-12-month-old child (OR = 1.87); living in a rural area (OR = 1.66); and male sex (OR = 1.53) (Table 5).

**Table 5:** Multivariate logistic regression model for ECC and S-ECC experience

Variables	ECC			S-ECC		
	^a^OR	^b^95% CI	^c^p	^a^OR	^b^95% CI	^c^p
Gender	1.48	1.07–2.05	0.018	1.53	1.06–2.20	0.023
Place of residence	1.90	1.36–2.65	≤0.001	1.66	1.16–2.40	0.006
Education level of parent	1.53	1.09–2.13	0.013			
Frequency of tooth brushing	1.41	1.01–1.96	0.044			
Over 12-month-old child bottle fed before or while sleeping	1.44	1.01–2.05	0.043	1.78	1.22–2.58	0.003
Over 12-month-old child fed with sweet beverages before or while sleeping	1.73	1.23–2.45	0.002	1.95	1.35–2.82	≤0.001
During the first 2 years of life child fed with sweet food	1.77	1.12–2.81	0.014	2.96	1.48–5.93	0.001

^a^OR – odds ratio based on logistic regression; ^b^CI – confidence interval for OR; ^c^p value

## Discussion

This survey’s purpose was to assess the strength of the association between the presence and absence of some well-known factors related to ECC in the Polish children at the age of 3 years. Our findings showed that ECC remains a great burden in this population, involving 53.8% of children and affecting 2.4 teeth. Its level however seems to stabilise, as within 13 years (2002 vs 2015) merely a slight diminution was found (caries prevalence from 56.2% to 53.8% and dmft value from 2.9 to 2.4).^29^ In Poland, dental care for patients until the age of 18 is financed by public health and is free irrespective of national health taxation of their parents/guardians. A child within his or her first 3 years of life can utilise the routine treatment of non-cavitated and cavitated carious lesions, endodontic and dental injuries treatment, fluoride varnish once a year, follow-ups three times a year, one visit for shaping a child’s behaviour to the dental setting as well as four preventive packages at the ages of 6, 9, 12 months and 2 years. However, such availability of dental care does not bring any expected beneficial outcomes. In contrast to the results, the implemented in Scotland’s (UK) national programme Childsmile provided reduction in obvious dental decay in 3-year-old children from 26% to 17% and dmft values from 1.1 to 0.4 within 4 years.^18^

Data regarding S-ECC at the age of 3 are rather scarce. The S-ECC prevalence of 6.5% has been reported in Lithuania^28^ and 44.1% in Northern Thailand.^24^ Nobile et al^20^ observed 2.7% of S-ECC among 36–71-month-old children from Southern Italy whereas 19% had ECC. In our population, S-ECC was recognised in 29.6% children. A great number of factors associated with ECC have been studied. However, not all of them reveal a statistically significant relationship with caries prevalence in a given population. Our data showed that the place of residence could influence the ECC development, as there was approximately 2-fold higher probability of ECC occurrence in rural area compared to urban one at bivariate and multivariate logistic regression analysis levels. The higher experience of ECC in children living in rural areas was also found in Brazil and China.^8,37^ The significantly higher prevalence of ECC in boys compared to girls has been presented in India^16^ and Latvia^12^ likewise in our study. Peltzer and Mongkolchati^24^ reported almost the same probability of S-ECC in boys (OR = 1.56) as our data at bivariate (OR = 1.21) and multivariate logistic regression analysis levels (OR = 1.53). In contrast to our finding, higher ECC prevalence in girls at the age of 3–6 from South India was noticed.^9^ No statistically significant difference between boys and girls was observed in Brazil^27^ and China.^37^

The level of parents’ education – especially the mother’s – was reported, as it is associated with caries development in children and the level of oral health-related knowledge, since parents have been mostly the main caregivers and oral health educators of their children. The lower ECC prevalence in children of mothers with a higher education level was demonstrated in some studies similarly to our data.^8,15,20,35^ Our study observed an association between a low level of dental health-related knowledge among parents and higher prevalence of ECC, confirmed by a previous report.^29^ The finding in our study that children with S-ECC were more likely to have visited a dentist could be explained by the higher number of caries-affected teeth (ie, higher dmft and dmfs values), probably leading to discomfort, toothache, infection or unaesthetic appearance. A similar observation was made by Nobile et al.^20^ Unexpectedly, despite offering free dental care for all children since their birth, in Poland so many as 48.0% (315/656) of the studied 3-year-olds had never visited the dental office so far.

It is well documented that oral hygiene practices are an effective measure in the prevention of caries development; however, in young children they should be performed or controlled by their caregivers. Preschool children do not have satisfactory manual dexterity to maintain good oral hygiene. Our data showed a statistically significant association of tooth brushing frequency with ECC occurrence but not with dmft value. Unexpectedly, we did not notice any association between child teeth cleaning by a parent and ECC prevalence similar to 4–5-year-old children brushing their teeth under adult supervision or not.^11^ In contrast, other studies presented a higher prevalence of ECC in children who practised tooth brushing by themselves in comparison to those who cleaned their teeth under parental supervision.^9,16,20^

A great number of variables related to feeding practices in young children such as duration of breast- and bottle-feeding, falling asleep during suckling or bottle-feeding, sweetened bottle content, sipping from the bottle during a day, snacking pattern of food containing fermentable carbohydrates have been studied.^5,8,9,14,16,20,23–25,28^ The WHO recommends exclusive breastfeeding for the first 6 months of life and then an introduction of appropriate complementary foods with continuation of on-demand breastfeeding until 2 years of age or beyond.^36^ A meta-analysis of cross-sectional studies showed that breastfed children were less affected by dental caries than bottlefed children.^3^ However, other systematic review and meta-analysis indicated that children breastfed after 12 months of age presented approximately 2-fold increase of caries risk compared to children breastfed shorter than 12 months, moreover children breastfed fed nocturnally or more frequently longer than 12 months revealed over 7-fold higher caries risk.^31^

The WHO recommends limiting the intake of free sugars to less than 10% of total energy intake in both children and adults (strong recommendation) and suggests a further reduction of the intake of free sugars to below 5% of total energy intake (conditional recommendation)^10^, ie, less than 16 g sugar for children aged 4–8 years.^2^ In the early childhood period, dietary habits and preferences for sweet taste are set with implications for future oral health.^6^ Therefore, the main advice for ECC reduction is no added sugar to food for children before the age of 2 years.^34^ According to the WHO guideline on sugars intake, dental professionals should be involved in providing their patients with eating advice regarding reduction of free sugars consumption (eg, to inform regarding content of free sugars in common foods and beverages, to encourage the consumption of fresh fruits and vegetables, nuts, seeds and wholegrain foods, to replace the drinking of sugary beverage by plain water and milk).^19^

In our study, the history of breast- or bottle-feeding and drinking sweet liquids over 12 months of age before or during sleep was associated with higher prevalence of ECC and S-ECC. However, the analysis of the relationship between duration of breastfeeding and ECC should be interpreted with caution since it is important to take into account that the study comprised children who were not breastfed at the time of examination. This may cause biased results. Feeding of children with sweetened food during the first 2 years of life caused over 2-fold increase of probability of ECC and almost 4-fold of S-ECC occurrence at bivariate level and approximately 2-fold of ECC and nearly to 3-fold of S-ECC in the final model of the multivariate logistic regression analysis.

Current frequent consumption of sweetened beverages and tea with sugar was also associated with higher occurrence of ECC and S-ECC as well as higher dmft values.

It is well known that starch-containing food can have a direct caries-inducing effect. Snacking potato crisps by children at the age of 1–4 was studied by Johansson et al.^14^ They found the statistically significant association between consumption of crisps and caries prevalence, ie, higher caries with intake of crisps. In contrast, our data did not show such a relationship with caries occurrence but only with dmft value.

The results of our study demonstrate that ECC affects a large proportion of 3-year-old children and is related to sociodemographic factors as well as behavioural and dietary habits, the current and the past. Therefore, factors promoting caries should be identified at the earliest possible age in order to implement proper home dental care. What is essential is that children must not consume any added sugars in their first 2 years of life.

ECC remains a statistically significant health problem due to its adverse effects on general health, physical growth and development, and oral health-related quality of life. Moreover, severity of ECC is a predictor for caries experience in permanent dentition.^26^ The WHO Expert Consultation on Public Health Intervention against Early Childhood Caries elaborated on a possible public solution to the ECC problem. It has been proposed that ECC prevention and control interventions could be integrated with the primary healthcare system. The management strategy should include primary (education of pregnant women, new mothers and caregivers regarding the common risk factors of ECC, ie, breastfeeding until 6 months of age, no added sugars for complementary feeding up to 2 years, and then the limited sugar intake, proper tooth brushing with fluoridated toothpaste), secondary (early detection of carious lesions and management, diet counselling, proper bottle-feeding, limited sugar intake, proper oral hygiene, check-ups) and tertiary prevention (reduction of the negative impact of untreated decay by treatment with use of ART, SMART and IRT).^26,34^ All of these actions should be strictly and consistently implemented in Poland.

The strengths of the study were: the children were recruited from the general population using a three-stage cluster sampling procedure, examined by calibrated dentists and their number was representative for the population. On the other hand, there were several limitations: questionnaires concerning factors influencing caries development, which were self-reported by parents, and the children covered by the survey were only those attending preschools.

## Conclusions

The most prominent risk factors for ECC were living in a rural area, consumption of sweetened foods within the first 2 years of age and nocturnal drinking of sweet beverages by the over-12-month-old child.
